# Pemphigus risk following programmed death receptor-1, programmed death ligand-1, or cytotoxic T-lymphocyte-associated protein 4 inhibitors: A 1:1 propensity-matched, global, retrospective cohort study

**DOI:** 10.1016/j.jdin.2025.01.004

**Published:** 2025-01-23

**Authors:** Justin Baroukhian, Kristina Seiffert-Sinha, Animesh A. Sinha

**Affiliations:** Department of Dermatology, Jacobs School of Medicine and Biomedical Sciences, 6078 Clinical and Translational Research Center, Buffalo, New York

**Keywords:** autoimmunity, bullous disease, CTLA-4 inhibitor, exposome, immune checkpoint inhibitor, immune-related adverse events (irAEs), PD-1 inhibitor, PD-1L inhibitor, pemphigus, trigger, TriNetX

*To the Editor:* Immune checkpoint inhibitors (ICIs), including programmed death receptor-1 (PD-1), programmed death ligand-1 (PD-1L), and cytotoxic T-lymphocyte associated protein 4 (CTLA-4) inhibitors, have significantly advanced cancer treatment by enhancing host antitumor immune responses.^1^ However, these agents can also precipitate immune-related adverse events (irAEs),^1^ including autoimmune conditions such as pemphigus, a potentially fatal blistering disorder characterized by autoantibodies against desmogleins. Despite scattered case reports suggesting an association, quantitative population-based evidence remains sparse.^2^, ^3^, ^4^ To address this gap, we leveraged the global federated health research network TriNetX to investigate the incident risk of pemphigus in patients treated with PD-1, PD-1L, or CTLA-4 inhibitors compared to a control group using electronic medical record data from over 120 million patients worldwide.

Within TriNetX’s Global Collaborative Network (accessed December 13, 2024), which integrates electronic medical records from >120 health care organizations, we defined two cohorts. Cohort 1 included patients treated with PD-1 inhibitors (pembrolizumab, nivolumab, cemiplimab), PD-1L inhibitors (atezolizumab, durvalumab, avelumab), or the CTLA-4 inhibitor (ipilimumab), and with no diagnoses indicating bullous pemphigoid (to reduce chances of miscoding/misdiagnosis). Cohort 2 comprised patients with general examination encounters (ICD-10: Z00), no evidence of exposure to any of the aforementioned ICIs, and no diagnosis of bullous pemphigoid. 1:1 propensity score matching using a greedy nearest-neighbor algorithm was employed to balance the cohorts for age, gender, race, and diagnoses indicating any malignancy (ICD-10: C00-D49). Demographic characteristics of cohorts 1 and 2 *after* propensity score matching are provided in Table I. The analysis encompassed risk assessment and Kaplan-Meier survival analysis, with outcomes measured from 1 day post index event to 730 days (2 years) (Fig 1).

**Table I:** Demographic characteristics after propensity score matching, outcome definition, and risk analysis details

Cohort 1 and Cohort 2 (each *n* = 152,795) demographic characteristics after propensity score matching		*P* = 1, standardized mean difference (SMD) < 0.001 for all demographic characteristics (*after* propensity score matching)∗
Age	64.9 ± 13.1 years	
Race		
White	64.2% (*n* = 98,053 per cohort)	
African American	7.1% (*n* = 10,834 per cohort)	
Asian∗		
Cohort 1	4.89% (*n* = 7437 per cohort)	
Cohort 2	4.615% (*n* = 7052 per cohort)	
Gender		
Female	41.7% (*n* = 63,700 per cohort)	
Diagnoses		
Any neoplasm, ICD-10: C00-D49	93.483% (*n* = 142,862 per cohort)	
Outcome	Presence of any of the following ICD-10 codes corresponding with various subtypes of pemphigus, *except* paraneoplastic pemphigus (L10.81), within 2 years of index event: L10.0, L10.1, L10.2, L10.3, L10.4, L10.5, L10.9, L10.89	

Cohort	Number of individuals with outcome†	Risk (%)	Risk (per 10,000 individuals)	Risk ratio (95% CI)	Odds ratio (95% CI)
1 (Exposed)‡	33/152,795	0.022	2.2/10,000	3.000 (1.516-5.936)	3.000 (1.516-5.937)
2 (Control)	11/152,795	0.007	0.7/10,000		

*ICD-10*, International Statistical Classification of Diseases, Tenth Revision, Clinical Modification.

∗ Difference for distribution of Asian individuals remained statistically significant (*P* = 0.0003, SMD = 0.0129), though of uncertain relevance given the small absolute differences relative to overall sample sizes.

† Excludes individuals with outcomes *prior* to index event.

‡ This includes individuals exposed to 1 checkpoint inhibitor or, possibly, a combination of multiple checkpoint inhibitors.

**Fig 1:**
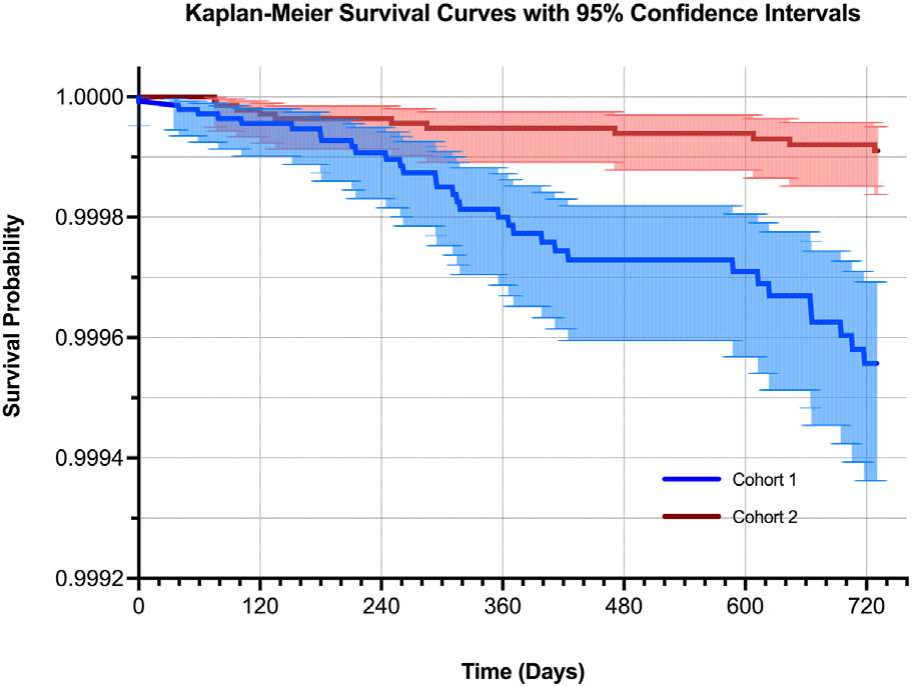
Pemphigus-free survival by Kaplan-Meier analysis among cohort 1 (ICI exposed) and cohort 2 (control) from 1 day to 730 days postindex event. Each line represents the proportion of individuals in a given cohort *without* pemphigus (pemphigus-free survival) following exposure to any ICI (cohort 1, *blue*) or from beginning of evaluation in controls (cohort 2, *red*) through 720 days (2 years) thereafter. Shaded areas represent 95% CIs. *ICI*, Immune checkpoint inhibitor.

The risk analysis demonstrated that the incidence of pemphigus was significantly higher in cohort 1 compared to cohort 2, with a risk ratio of 3.000 (95% CI: 1.516-5.936; *P* = 0.0009) and an odds ratio of 3.000 (95% CI: 1.516-5.937) (Table I).

The Kaplan-Meier survival analysis further corroborated these findings, showing a significantly higher hazard in cohort 1 (hazard ratio 4.651, 95% CI: 2.34-9.246) (Fig 1). These results underscore a significant increase in pemphigus risk among patients treated with PD-1, PD-1L, and CTLA-4 inhibitors, suggesting that ICIs may induce autoimmune responses through mechanisms such as immune dysregulation or regulatory T cell depletion.^1^^,^^5^

This investigation, leveraging the extensive dataset and propensity-matched design of the TriNetX platform, provides robust evidence for an association between ICIs and pemphigus risk. Nevertheless, limitations resulting from the retrospective nature of the study (including the inability to establish causality), computational constraints of the TriNetX platform (particularly those related to the parameters of the propensity score matching process), and potential residual confounding factors should be acknowledged. Future research, including prospective studies and mechanistic investigations, is necessary to elucidate the biological processes underlying this association and to develop targeted interventions for at-risk patients.

Clinicians prescribing PD-1, PD-1L, or CTLA-4 inhibitors should be aware of the increased risk of pemphigus and monitor patients closely for early signs of this condition. Integrating detailed clinical assessments and patient education will be essential in effectively managing this risk.

## Conflicts of interest

None disclosed.
